# Social ties in old age: the effect of the COVID-19 pandemic

**DOI:** 10.1007/s10433-025-00889-3

**Published:** 2025-11-04

**Authors:** Ashira Menashe-Oren, Damiano Uccheddu, Ester Lucia Rizzi

**Affiliations:** Université Catholique de Louvain, Ottignies-Louvain-la-Neuve, Belgium

**Keywords:** Social networks, COVID-19 pandemic, Older adults, SHARE

## Abstract

Social ties amongst older adults were immediately affected by the COVID-19 pandemic—both by the deaths occurring disproportionately amongst older adults and by the policies limiting social contact implemented by governments to curb the spread of the virus. We explore changes in close social networks amongst older-aged adults before and during the pandemic across 13 European countries using panel data from the Survey of Health, Ageing and Retirement in Europe. We utilise four waves of data collected over a decade, the latest of which was during the COVID-19 pandemic in 2021–2022. The social network is measured based on respondents’ reports on confidants, individuals with whom respondents discuss important matters, whether in-person or remotely. Results from individual fixed and random effects models indicate that while it appears that the number of confidants older adults have is not associated with the pandemic, the churning of these confidants was considerable. Older-aged adults lost members of their close network much more over the pandemic period than they did beforehand, though new social ties were also made. Across all waves, and especially over the pandemic, we find significant instability of social resources, which could have important implications for older adult well-being.

## Introduction

Social networks, defined as the interconnected web of social relationships surrounding an individual (Mcpherson et al. 2005, 2009), change substantially over the life course, as new relationships are formed while others fade or are lost (Wrzus et al. 2013; Antonucci et al. 2014). As people age and become more aware of the limited time they have left, they tend to prioritize relationships of high emotional quality, focussing on close relations (Lang et al. 1998; Carstensen et al. 1999; Schwartz and Litwin 2018). Changes in the social networks of older-aged individuals are especially important to consider as societies age. In Europe, the proportion of over 60-year-olds in 2021 was 26% of the population and is expected to increase to 36% by 2050 (United Nations 2022).

Having a social network is an important resource at older ages. It is important for health and well-being (Kriebs 2024), and is a predictor of longevity (Eriksson et al. 1999). Such effects may differ according to who is part of the network (Berkman et al. 2000)—a spouse, friends, family, or others (Fiori et al. 2007, 2008). Amongst older adults, social networks can provide a source of care, including home maintenance and transport, and emotional support (Himes and Reidy 2000; Barker 2002; Antonucci et al. 2014; Batur et al. 2024). Social networks also provide security, buffering against the adverse consequences of crises. Therefore, during the COVID-19 pandemic, social networks were a critical resource, but their protective role was constrained by policies limiting face-to-face contact.

The effects of the COVID-19 pandemic on social ties have only partially been interrogated. Yet investigating the effects of the pandemic on social networks is imperative in preparing for future crises. The pandemic was an exogenous shock, unforeseen and irreversible, with the potential for dramatic changes in social networks—whether weakening or strengthening ties—and for threatening or reinforcing the social fabric of society. In this article, we focus directly and uniquely on social networks, examining *changes* over the pandemic as compared to earlier years. How did the pandemic shock affect the social networks of men and women in older ages?

We examine the COVID-19 pandemic effects on the social networks focussing on older adults since they were most vulnerable to the virus and disproportionately affected by COVID-19. We use harmonized cross-national panel data to examine the (in)stability of social networks among adults aged 50 and over before and during the pandemic in Europe. The social network is assessed based on reports on who they discuss important things with, confidants. No distinction is made between in-person and remote relations, since the tie is qualified as important independent of how the relation is maintained. We compare reports on social ties pre-pandemic, from 2011 to 2019, with reports from 2022 covering the pandemic period. We focus on the (in)stability of social networks, specifically the evolution of size and composition, and losses and gains of the confidant network. The confidant network is defined as the group of individuals with whom respondents discuss important matters, either in-person or remotely. Of existing literature on social network changes during the pandemic, this study is the first to jointly examine these characteristics of social networks: turnover, composition, and size. Assessing only network size provides an incomplete picture by masking network stability or instability. By examining gains and losses of confidants we offer a more extensive and nuanced understanding of the social experience of older adults. Moreover, this study moves beyond previous work, which has largely relied on cross-sectional and single-country analyses, by taking a longitudinal, cross-national comparative approach to provide a comprehensive perspective on how older adults’ social networks can be reshaped during a global crisis.

### Changes in social networks at the time of the COVID-19 pandemic

The direct effects of the COVID-19 pandemic and the associated policy measures taken to contain the spread of the SARS-CoV-2 virus contributed to immediate changes in social ties. Social networks amongst older adults at the time of the pandemic were altered by overall higher death rates (Msemburi et al. 2023). Simply put, people lost members of their social network because they died. Since the virus disproportionately affected older adults in terms of mortality (O’Driscoll et al. 2021; Torres et al. 2023), older individuals were more likely to lose spouses and friends—close members of their social network. In addition to increased mortality, many older adults were also left in poorer health during the pandemic due to changes in access to healthcare (Arnault et al. 2022; Tavares 2022).

Policy restrictions limiting social ties, such as the closure of schools and offices or stay-at-home orders, further had an impact on both the frequency of social interactions and network size. In the United States, between a third and a half of older adults experienced a decline in in-person contact with family and friends who did not live in the same household (Choi et al. 2022), with weekly contact declining by around 20% over the pandemic (Freedman et al. 2022). Similarly, network size was reduced over the pandemic by close to 50% (Haggerty et al. 2023). One study in Europe found that, amongst all ages, the number of social contacts outside of the home decreased during the pandemic due to governmental restrictions; in Italy for example, the average number of daily contacts pre-pandemic was around 20, but during the pandemic ranged from 2.2 to 3.7 (Wong et al. 2023). Likewise, when it comes to the closer social network and individuals with whom personal matters are discussed, a decrease from 3.8 confidants to 0.4 was noted during the first three months of the pandemic in Canada (Bierman et al. 2021). Moreover, in Hungary, declines in in-person interaction were mostly noted among adults aged 60 and older (Dávid et al. 2023).

All the same, there is some evidence of stability of social contacts during the pandemic. A study in Germany found that, during the lockdowns, social ties amongst adults of all ages were relatively stable for more than half of the study respondents. Overall, however, individuals lost more friends and acquaintances than they gained, especially women (Bertogg and Koos 2022). Digital technology appears to have contributed to preserving social ties among older adults during the pandemic (Sixsmith et al. 2022; Schafer 2024). In the U.S., most older adults maintained regular contact with non-co-resident family or friends via the phone or messaging, although only a small share increased the number of contacts remotely (Hawkley et al. 2021; Freedman et al. 2022).

Some social ties become stronger, filling social contact vacuums  while abiding to policy restrictions. In particular, family and friends were found to play a role in helping to cope with adverse economic conditions, and in giving emotional and instrumental support at the time of the pandemic (Coleman et al. 2022; Panarello and Tassinari 2022). This is reflected in the increased proportion of family ties within social networks (Dávid et al. 2023). Moreover, in North America and the United Kingdom, the quality of social connections improved during lockdowns when individuals lived with partners (Okabe-Miyamoto et al. 2021).

Finally, spouses maintain a central role in social networks (McLaughlin et al. 2010; Nakash et al. 2022; Sun and Schafer 2023), and this was also confirmed during the pandemic. During this period, there was evidence of an increase in the quality of marital relationships (Vanterpool et al. 2025), likely due, at least in part, to the greater difficulty of maintaining social ties outside the household.

### Gendered social networks

Gender can shape both the structure and function of social networks throughout the life course, including in later life. Women generally maintain larger, more varied networks, with less family-centric ties, stronger support systems, and more frequent interactions than men (Antonucci et al. 1998; Cornwell et al. 2009; McDonald and Mair 2010; Fischer and Beresford 2015; Santini et al. 2016; Ali et al. 2022). Women also engage more in formal and informal social activities (Cornwell et al. 2008; Finkel et al. 2018), and expand their networks over time, even as family involvement decreases (Schwartz and Litwin 2018). Moreover, women’s social networks are also more closely associated with subjective well-being (Pinquart and Sörensen 2000). In contrast, older men’s networks are often more spouse-centred (McLaughlin et al. 2010; Nakash et al. 2022), placing them at greater risk of social isolation when they lose their partner (Litwin and Stoeckel 2014). Indeed, spouse-exclusive networks consist of over a quarter of social networks in older ages, and make men in particular more vulnerable when losing a spouse (Sun and Schafer 2023). The accumulation of risk factors including widowhood and health decline renders older men more susceptible to the detrimental social consequences of the COVID-19 pandemic. Nevertheless, some results suggest a greater disadvantage for women. In the Netherlands, having a spouse/partner was found to be particularly important during the pandemic for support when ill (Steijvers et al. 2023), suggesting that women, who are more likely to be widowed and not to have partners in older ages, were more vulnerable. Finally, additional factors likely influenced how men and women differentially experienced changes in their social networks over the pandemic. One such factor is the gendered difference in caregiving responsibilities, with women bearing greater burden even in older ages (Spatuzzi et al. 2022). Another factor is a gender gap in digital literacy, with women’s disadvantage increasing with age (Bachmann and Hertweck 2025).

### Variation in social networks across countries

Social networks in later life are shaped not only by individual characteristics, but also by the broader society, including country-specific conditions, such as socio-cultural norms, economic conditions, and welfare structures (Nyqvist et al. 2019; Seifert and König 2019; Heu et al. 2021), as well as the level of trust in public institutions (OECD 2021; Rapolienė and Aartsen 2022). These macro-level factors can constrain or enable individual engagement in social networks, influencing both the availability of social ties and the normative expectations around maintaining them.

The structure and functioning of older adults’ social networks, including formal and informal ties, are closely linked to welfare institutions (Nyqvist et al. 2019, 2025). Universal and generous social rights foster conditions that promote social participation, potentially reducing reliance on (informal) social networks for addressing social risks. Social Democratic countries, such as Denmark and Sweden, maintain a universalistic and all-encompassing approach to welfare (Esping-Andersen 1990). Similarly, Bismarckian welfare states, such as Austria, Belgium and France, tend to have lower levels of dependency on personal social networks for the provision of care and support (Craveiro 2017), and higher social integration among older adults (Nyqvist et al. 2019). In contrast, Southern European countries like Italy and Spain, and some Post-Socialist welfare states, such as the Czech Republic and Estonia, are characterized by weaker social protection and stronger reliance on family support (Ferrera 1996; Bob 2000), which is associated with higher levels of social isolation and vulnerability among older populations (Craveiro 2017; Nyqvist et al. 2019).

These pre-existing institutional differences were reflected in distinct policy responses to the COVID-19 pandemic, particularly regarding the stringency of lockdowns and the provision of targeted support for older adults. Social Democratic and some Bismarckian countries implemented more targeted and less stringent measures. Sweden, for example, avoided formal lockdowns, while Denmark and Switzerland adopted comparatively milder restrictions (Eurofound 2022; Dones and Ciobanu 2024). Social Democratic, and to a lesser extent some Bismarckian countries, extended existing universal welfare policies, buffering socioeconomic inequalities and maintaining their institutional logic during the COVID-19 pandemic (Pereirinha and Pereira 2021; Ellison et al. 2022). In contrast, Southern European and several Post-Socialist welfare states, with weaker social infrastructure and limited public support for older adults, enforced very strict, prolonged lockdowns (Pereirinha and Pereira 2021; Caro et al. 2022; Eurofound 2022).

Many Post-Socialist states carry a historical legacy of totalitarian rule, which shattered trust in public institutions. This legacy had consequences for loneliness, which have persisted decades later, particularly among older cohorts who lived most of their lives under these regimes (Rapolienė and Aartsen 2022). The effectiveness of COVID-19 policy responses may have been influenced by such pre-existing levels of institutional trust (OECD 2021; Eurofound 2022). In high-trust contexts, public health advice was more likely to be followed, and state-supported initiatives more likely to be utilized. In low-trust contexts, scepticism towards public guidance may have led to lower compliance, reluctance to use formal support services, and increased loneliness (OECD 2021; Rapolienė and Aartsen 2022). This may have intensified pressure on already fragile informal networks (Nyqvist et al. 2019). Critically, the type of policy response had variable effects on older people’s social lives. Stricter lockdowns were associated with greater social isolation and loneliness among older adults across Europe (Caro et al. 2022). Nonetheless, many countries actively worked to offset these effects through targeted support such as meal delivery services, digital inclusion programs and increased paid leave for informal carers (OECD 2021; Eurofound 2022). In countries with strong stringency policies, limited formal support and low institutional trust, older adults were vulnerable to sharp disruptions in social engagement. This combination created a precarious paradoxical situation: where state support was needed due to stricter lockdowns, it was least effective due to low institutional trust. In such situations, the pandemic likely strained informal care arrangements, leading to higher network churn, as traditional support systems were disrupted (Litwin and Stoeckel 2014).

### Hypotheses

In light of these contextual differences in shaping social networks, gender diversity in social networks and previous studies looking at the change in social networks at the time of the COVID-19 pandemic, we evaluate five sets of hypotheses.

First, we expect stability in confidant network size during the crisis because of the importance of close ties. Moreover, social networks in later life are considered stable and less prone to turbulence (Wrzus et al. 2013; Weiss et al. 2022), meaning they should remain strong and survive the pandemic shock. Moreover, communication technologies allowed for maintaining social ties. We hypothesize that older adults experienced stability in the size of their confidant networks during the pandemic (H1a). That said, given that the policies at the time of the pandemic imposed structural constraints, like mobility restrictions, this limited opportunities to maintain or expand social ties (Wong et al. 2023). Moreover, the pandemic reduced the social network of older adults because they lost companions who died from the SARS-CoV-2 virus. We therefore expect the pandemic to have disrupted this upward trend, resulting in a decline in confidant network size among older adults (H1b).

Second, since confidant network size may offer only partial insight into the underlying relational dynamics, we consider churning of the network. Network size may appear stable because losses of confidants are compensated by gains of confidants. Such (in)stability of networks can be operationalised as a change in the number of confidants gained or lost. We expect that the pandemic intensified the turnover of social ties due to physical distance policies, emotional strain and changes in daily interaction patterns (Thoresen et al. 2021). We hypothesize that with the restrictions on social contacts, older adults experienced a higher intensity of lost ties during the pandemic than in previous periods (H2). At the same time, strengthened household ties and increased use of digital technologies may have facilitated the formation or reactivation of social ties (Okabe-Miyamoto et al. 2021; Schafer 2024). Existing social ties could have been redefined as stronger ties. Therefore, despite the constraints of the pandemic policies, we hypothesize that older adults also experienced an increase in the gain of new confidants during the COVID-19 pandemic (H3). Overall, due to increased losses and gains, we expect greater instability of social ties during the pandemic compared to the pre-pandemic period.

Fourth, the composition of networks may have changed, especially in relation to spouses, given the smaller size of social networks in older ages and the prioritization of emotionally close and proximate ties in later life (Carstensen et al. 1999; Schwartz and Litwin 2018). We hypothesize that spouses remained central members of older adults’ social networks during the COVID-19 pandemic (H4a). Nonetheless, given the heightened risk of partner death at the time of the pandemic (Wang et al. 2022a), and potential strains on partner relationships (Bellani and Vignoli 2022), which may have altered the role of the spouse in the network, we hypothesize that the inclusion of spouses in older adults’ confidant network declined during the pandemic (H4b).

Finally, the interplay between welfare state characteristics, the stringency of COVID-19 policies, and levels of institutional trust leads us to expect considerable heterogeneity in social network outcomes across the European countries in this study. We hypothesize that the instability of older adults’ social networks during the COVID-19 pandemic varied across countries according to welfare state characteristics, policy stringency, and institutional trust (H5). We expect that older adults in low stringency, high support and high trust countries, like Denmark and Switzerland, experienced the least disruption to their social networks, while those in high stringency, low support and low trust countries, like Italy and Poland, experienced the greatest disruption.

## Data and methods

### Survey of Health, Ageing and Retirement in Europe data

We utilise data from the Survey of Health, Ageing and Retirement in Europe (SHARE), available for download on request (Börsch-Supan et al. 2013), which are advantageous in being longitudinal and cross-national.^1^ SHARE is a panel study focussing on older-aged adults, and has a Social Network module included in four waves of data collection: wave 4 in 2011, wave 6 in 2015, wave 8 in 2019-2020, and wave 9 in 2021-2022.^2^ The surveys in these years collected data through face-to-face interviews using computer-assisted personal interviewing. Twenty-eight countries participated in this module, though not all countries feature in all waves. We focus solely on countries participating in all four waves, thirteen in total, to provide a longitudinal perspective, capturing social networks before and during the pandemic.^3^ Together, the surveys cover 85,505 individuals across countries, with respondents’ median age of 67 and 57% female (Table 1). We use a cross-country perspective to capture the variation in contextual differences in social networks across countries (types of welfare states, different cultures etc.), and especially considering the variation in COVID-19 stringency policies. As evident in Table 1, policies meant to curb the spread of the SARS-CoV-2 virus such as school closures, cancellation of public events, and reduced public transport were not yet introduced in the post-Socialist states of Slovenia and Estonia as of March 1 2020, while in Bismarckian France and Italy, the stringency index was already high. Similarly, two years later, some countries maintained strong policies, such as Bismarckian Austria and Italy, while others had looser restrictions, as in Universalist Denmark, Sweden and Switzerland.

**Table 1:** Countries with SHARE social network modules, respondents’ characteristics, and pandemic-related information

Country	Number of SHARE respondents in total	Proportion of respondents in all four waves	Proportion of respondents with missing social network data^*^	Age and gender composition of SHARE respondents		COVID-19 stringency policies^#^		Excess mortality	
				Median age	Proportion female	1 March 2020	1 March 2022	1 March 2020	1 March 2022
Austria	6,588	24.5%	1.47%	68	58.6%	11.11	47.79	− 3%	1%
Belgium	8,678	22.2%	6.66%	65	55.8%	11.11	29.79	− 12%	− 8%
Czech Republic	7,779	25.4%	0.80%	68	60.1%	19.44	36.15	− 4%	0%
Denmark	4,654	27.5%	2.75%	66	54.3%	11.11	13.89	− 13%	8%
Estonia	8,450	32.3%	4.66%	68	61.3%	0	45.3	− 6%	29%
France	7,415	35.6%	2.91%	67	57.5%	34.72	29.85	− 7%	− 1%
Germany	7,090	14.2%	0.58%	67	53.5%	25	38.65	− 11%	− 5%
Italy	6,755	18.5%	10.13%	68	55.7%	64.35	43.78	− 1%	− 1%
Poland	6,210	15.8%	4.31%	67	56.6%	11.11	22.53	− 4%	12%
Slovenia	6,676	22.2%	10.16%	67	56.2%	0	14.26	− 6%	3%
Spain	7,350	19.9%	3.61%	69	56.0%	11.11	38.39	− 8%	− 5%
Sweden	4,854	21.7%	0.66%	71	54.2%	5.56	15.5	− 9%	− 2%
Switzerland	4,194	45.2%	0.32%	67	55.1%	19.44	14.81	− 11%	− 4%

*The proportion of respondents with missing social network data refers to those who did not answer the social network module, for whom we have no information on the network at all. There may be missing information on specific social network indicators such as confidant’s relationship to respondent, but these are not included here. ^#^The Stringency Index is based on nine metrics: school closures, workplace closures, cancellation of public events, restrictions on public gatherings, closures of public transport, stay-at-home requirements, public information campaigns, restrictions on internal movements and international travel controls (Hale et al. 2021). A higher index indicates a stricter response, with the maximum being 100. Excess mortality is measured using a P-score which is the ratio of excess to expected mortality for all ages, expressed as a percentage (Msemburi et al. 2023). Higher P-scores indicate higher levels of excess mortality. Data for excess mortality and stringency policies are available at: https://ourworldindata.org/search?q=covid

The SHARE data we use spans just over a decade, covering people over age 50. As such, it is likely that some respondents died between waves. Similarly, respondents may tire of participating in the panel survey and refuse to continue, or may migrate and not be traced. Loss to follow-up is therefore important to deal with. Samples were refreshed between waves to be representative and cover a sufficiently large population. Overall, the proportion of respondents interviewed in all waves, and who responded to the social network module of the questionnaire, is relatively low, ranging from only 14% of respondents in Germany to 45% in Switzerland (Table 1). We account for sample attrition in our modules (explained further below). Moreover, we test for the robustness of our results by analysing only respondents who were interviewed in all waves (25% of respondents).

### Measuring the social network

The SHARE questionnaire includes a direct subjective method to identify social networks, asking: “*Over the last 12 months, who are the people with whom you most often discussed important things.?*” 4 This is aimed at identifying confidants with whom respondents share personal details and discuss important issues (McPherson et al. 2009; Litwin and Stoeckel 2014). Such a name generator is a well-established survey method to map social networks (Marsden 1990; Cornwell et al. 2009). Respondents can name up to seven confidants, family members, and non-kin ties (such as friends, neighbours, and caregivers) alike, providing characteristics of these social network members including age, gender, and relationship to the respondent. Although the size of the network is limited to seven, across countries and years, the mean number of listed confidants is 2.4 for men (with a standard deviation of 1.6 and median of two) and 2.8 for women (with a standard deviation of 1.7 and median of three) (Table 2). In using this SHARE social network data, we implicitly refer to social ties that are meaningful to the respondents. While the self-reported name generating module allows us to map out part of the social network of respondents, it is important to note that people do not always confide in individuals with whom they have strong ties (Small 2013; Small et al. 2024). The social network we examine is of confidants only, whether strong or weak ties, family or non-kin.^5^

**Table 2:** Descriptive statistics (mean and standard deviation) of key outcome variables, measures of social network, across waves

Country	Gender	Network size	Proportion of respondents with spouse in network	Number of lost confidants	Number of new confidants
Austria	Male	2.76 (1.68)	0.77 (0.42)	2.08 (1.30)	1.27 (1.40)
	Female	3.12 (1.69)	0.52 (0.50)	2.11 (1.26)	1.39 (1.39)
Belgium	Male	2.65 (1.76)	0.63 (0.48)	2.23 (1.40)	1.57 (1.54)
	Female	3.07 (1.78)	0.43 (0.50)	2.28 (1.37)	1.63 (1.52)
Czech Republic	Male	2.10 (1.40)	0.74 (0.44)	1.81 (1.06)	1.05 (1.26)
	Female	2.44 (1.47)	0.46 (0.50)	1.88 (1.12)	1.21 (1.30)
Denmark	Male	2.58 (1.59)	0.77 (0.42)	2.01 (1.22)	1.30 (1.38)
	Female	3.27 (1.57)	0.62 (0.48)	2.14 (1.30)	1.55 (1.47)
Estonia	Male	2.02 (1.41)	0.74 (0.44)	1.84 (1.12)	0.98 (1.27)
	Female	2.52 (1.52)	0.42 (0.49)	1.93 (1.16)	1.13 (1.33)
France	Male	2.52 (1.65)	0.62 (0.48)	2.16 (1.31)	1.55 (1.49)
	Female	2.87 (1.68)	0.40 (0.49)	2.21 (1.32)	1.59 (1.47)
Germany	Male	2.83 (1.58)	0.79 (0.41)	2.07 (1.28 )	1.51 (1.48)
	Female	3.28 (1.60)	0.63 (0.48)	2.19 (1.32)	1.68 (1.52)
Italy	Male	2.08 (1.40)	0.76 (0.42)	1.81 (1.12)	0.96 (1.18)
	Female	2.29 (1.41)	0.55 (0.50)	1.82 (1.07)	1.06 (1.20)
Poland	Male	2.00 (1.43)	0.75 (0.44)	2.00 (1.30)	1.46 (1.49)
	Female	2.32 (1.49)	0.53 (0.50)	2.22 (1.38)	1.61 (1.50)
Slovenia	Male	2.09 (1.54)	0.78 (0.42)	2.05 (1.37)	1.40 (1.52)
	Female	2.42 (1.57)	0.56 (0.50)	2.08 (1.35)	1.51 (1.49)
Spain	Male	2.31 (1.57)	0.74 (0.44)	2.01 (1.29)	1.29 (1.43)
	Female	2.59 (1.62)	0.54 (0.50)	1.95 (1.22)	1.37 (1.39)
Sweden	Male	2.71 (1.64)	0.74 (0.44)	2.06 (1.29)	1.44 (1.49)
	Female	3.39 (1.75)	0.55 (0.50)	2.24 (1.38)	1.65 (1.53)
Switzerland	Male	2.67 (1.64)	0.76 (0.43)	2.15 (1.32)	1.24 (1.41)
	Female	3.07 (1.69)	0.56 (0.50)	2.17 (1.33)	1.29 (1.37)

The top number in each cell is the mean, and the bottom number in parenthesis is the standard deviation. The maximum network size, number of lost or gained confidants is capped at seven.

**Fig. 1:**
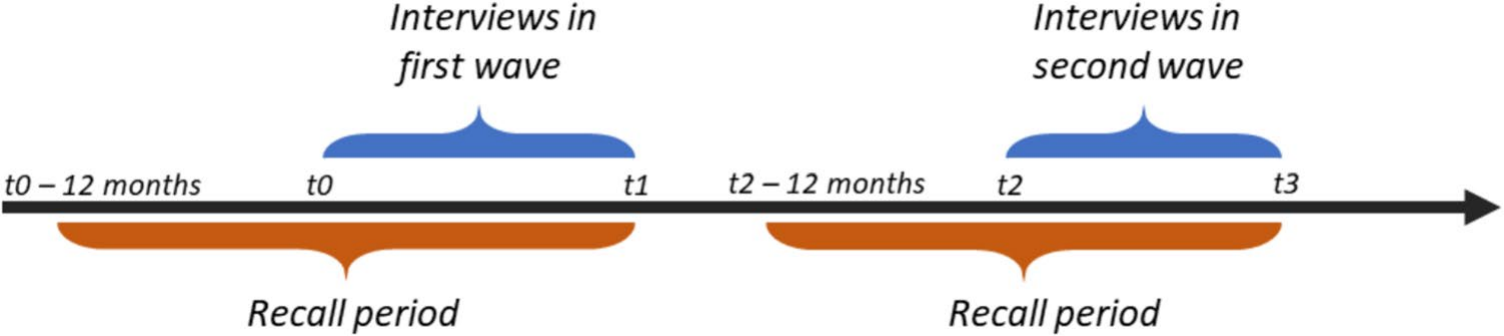
Schematic depiction of data collection and the timing of interviews

Since respondents were asked to specify their confidants in the last 12 months, this recall period can vary according to the time of the interview. In the schematic depiction of the timing of interviews and of the recall period in Fig. 1, it is clear that the recall period, aggregated, covers a wider time frame than the time of data collection itself. An individual interviewed early on during the data collection period will have a recall period that extends back further away from the time data collection began. In contrast, for an individual interviewed towards the end of the data collection period, their recall period mostly covers the time of data collection. Moreover, the gap between the interviews could be potentially large if the individual was interviewed early during the first wave and late during the second wave. In our analysis, we refer to the timing of the surveys according to when the bulk of interviews were held: in wave 4, 2011, in wave 6, 2015, in wave 8, 2019, and in wave 9, 2022 (see Appendix Fig. 8), referring to all of the data collection period between t0 and t1 for example (Fig. 1). Nonetheless, it is important to note that the reports on confidants reflect the respondent’s network at the time of the survey and up to twelve months before. This is particularly important for wave 9, where the recall period can extend back to October 2020, when the pandemic was in full swing and excess mortality was high (Msemburi et al. 2023), and wave 8, where the last interviews were in March 2020 and the recall period was largely before the start of the pandemic.^6^ All the same, recall is typically more reliable for the time closest to the interview.

The SHARE data is cleaned and harmonized across countries and waves, and variables based on original questions from the interview are generated by the SHARE project team. Missing values due to item nonresponse and errors are dealt with by SHARE through hot-deck imputation^7^ when a negligible fraction of values are missing (SHARE-ERIC 2024). The proportion of missing values, that is, non-response on the social network module, is relatively low (see Table 1). Of those with missing data, 53.4% were women, and 54% were between ages 50 and 64 (as compared to 57.1% of respondents without missing values who were women, and 37.9% in the younger age group). The highest proportions of missing data, 10% of respondents, were in Italy and Slovenia. Generated variables we rely on include network size and the number of new or lost members of the social network since the last wave of data collection. Following wave 4, when respondents are asked to name their confidants, the list of people named is compared to who they listed in the previous wave (SHARE-ERIC 2024). By comparing these lists (confidants reported on referring to the two recall periods as demonstrated in Fig. 1), it is possible to examine the changes in each respondent’s social network. Since the social network module used in SHARE is a multiple-name generator, the risk of mismeasurement of lost and new ties is reduced (as compared to single-name generators) (Marin and Dubash 2021).

Variables of new or lost confidants counts the number of confidants who were either not mentioned last time (in the previous wave) but mentioned now, or not mentioned this time (in the current wave) but were mentioned previously. As such, these variables trace changes between waves. When a respondent does not name someone previously named, they are asked an additional question about why this person is no longer a confidant. One potential reason for losing a confidant is the death of the confidant; thus, in examining the reasons, we are able to identify whether the pandemic affected social networks through mortality. We further examine who new confidants are, in terms of their relationship to the respondent, allowing us to consider whether new ties made during the pandemic period were of a certain type.

### Modelling approaches

Our analysis is country-specific to capture the variation across countries in culture and in social values, as well as the differences in policies implemented to contain the coronavirus, such as cancellation of public events and closure of workplaces, which may differentially affect social networks and/or the variation in excess mortality across countries (see Table 1). Moreover, since social networks are culturally and state defined, there is likely variation across countries. We model each country separately to ensure country-specific effects are not balanced out or muted by being pooled together. In addition, our analysis is gender-specific, since networks differ between men and women (Schwartz and Litwin 2018).

In examining social networks amongst older adults before and during the pandemic, we use descriptive analysis and random and fixed effects models,^8^ using STATA 18. For descriptive analysis, we use sample weights. Since it is possible that respondents dropped out of the survey at some point, we account for panel attrition using inverse probability weights of dropping out of the panel (regardless of reason – death, migration, non-response, etc.) (Jones et al. 2013). Weights were created by identifying the censored respondents and predicting the probability of attrition for each wave separately.^9^ Using this method, we adjust for attrition by assigning greater weight to individuals with key demographic, socioeconomic, and health characteristics associated with a high probability of dropping out of the panel.

The outcome variables we use in our models are (1) network size, (2) the presence of a spouse in the network, (3) the number of network members lost, and (4) the number of new network members, as described in Table 2. Respondents who only feature in one wave are excluded from models of new and lost members (outcomes 3 and 4), since changes in social networks cannot be observed. All models are stratified by country and gender, with respondent random and fixed effects.^10^ Thus, we run 26 models per dependent, altogether 94 models (four outcomes × 13 countries × two genders). To synthesize our country- and gender-specific results, we compare predicted marginal effects using plots, assuming all covariates apart from wave are held at their mean.

Wave, or time period covered by survey, is our key independent variable. We chose to use this rather than the exact year or date of interview to get an aggregated perspective of the pandemic effect. As such, when we refer to wave 9, or 2022, we refer to the twelve months extending back from each interview for which confidants were reported on, covering the period between October 2020 and September 2021, very much during the thick of the pandemic (see Fig. 1 and Appendix Fig. 8). In addition to wave, covariates included in the models are only time-varying: age group of the respondent (50–64, 65–79, and 80 + year olds), residence (rural, town, city), retirement status, and number of chronic illnesses (as a general measure of health status).^11^ In models of lost and new members, we also include a covariate of the size of the network. Residence, retirement and health status all account for the possibility of having more or fewer people in one’s social network due to circumstances increasing or reducing the potential frequency of contact with others (Vacchiano et al. 2024). We used a stepwise approach in testing the effect of each covariate before finalizing our model.

## Results

### Increasing number of social ties

**Fig. 2:**
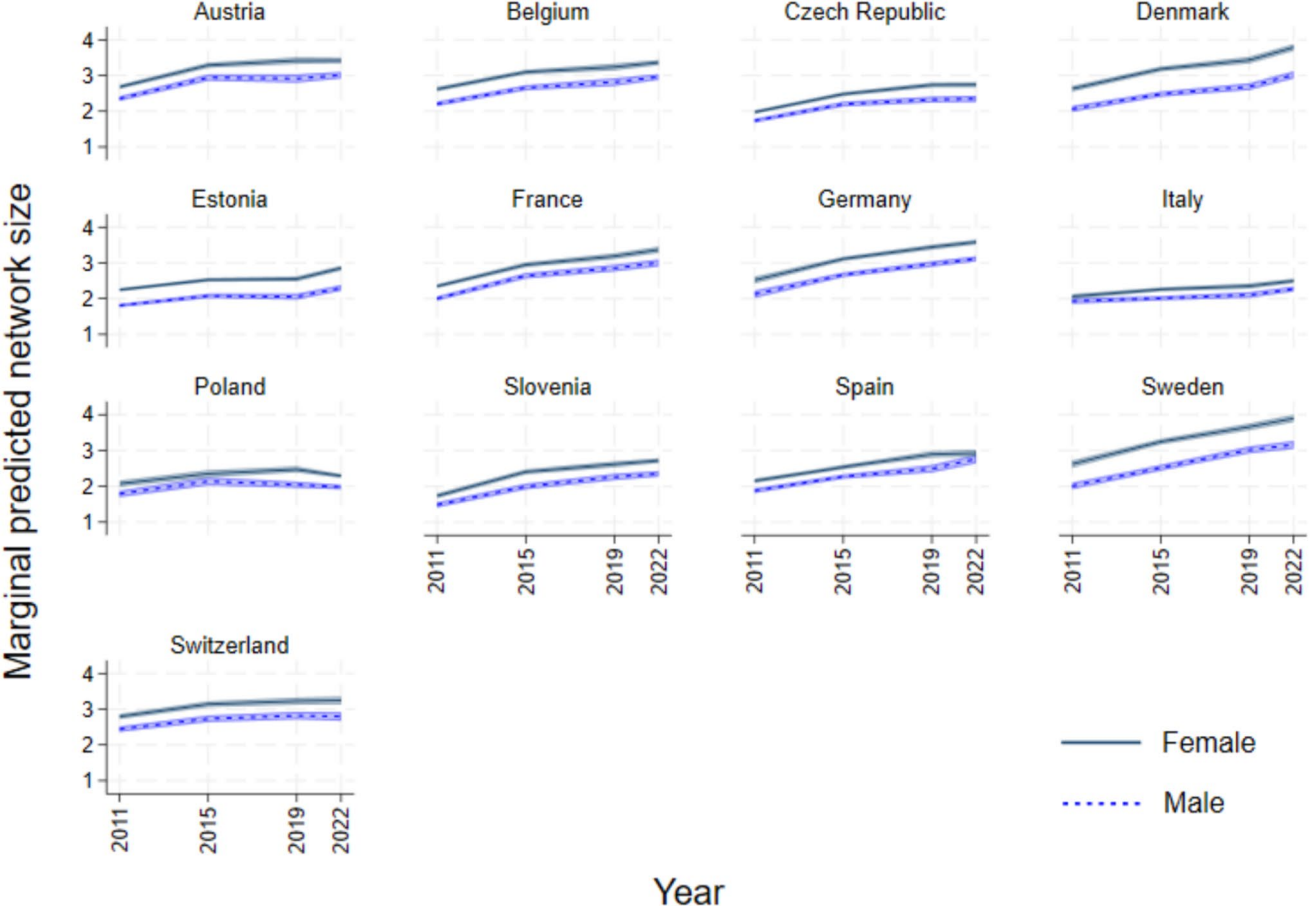
Predicted margins of social network size based on country- and gender-specific mixed models accounting for panel attrition

The mean number of social ties amongst older adults is greater amongst women, as evident in Table 2, ranging from 2.24 in Italy to 3.39 in Sweden (compared to a low of 2 in Poland and a high of 2.83 in Germany amongst men). Social network size evolves over time, but this gender gap remains quite steady, as evident by the non-overlapping confidence intervals in most countries as seen in Fig. 2. In some countries, as in Italy, Spain and to some extent Poland, there are negligible differences in network size between the genders, and the mean size is generally smaller.

According to our set of country- and gender-specific models, predicting network size (Fig. 2), as compared to 2011, older adults saw a gradual increase in the number of confidants over time. In some countries, this even doubled, as in Germany, Slovenia, and Sweden. In Denmark and Estonia, there was a clear positive and statistically significant shift in trends over the pandemic period. Additionally, in Denmark, women’s social networks grow more than men’s. In Austria, Italy, Spain and Switzerland, we find that increasing network size appears to stall over time, especially during the pandemic period. In Poland, the size of women’s social networks even declined over the pandemic. Overall, a statistically significant increase in network size occurred between 2011 and 2015 (from closer to two to closer to three confidants), and in many countries also between 2015 and 2019. In the pandemic period, we observed variance across countries. This general earlier increase in network size that, in most cases, tapers during the pandemic is also found when we model only respondents who participated in all four waves (Appendix Fig. 9, panel a).

### The effect of the pandemic on losing confidants

**Fig. 3:**
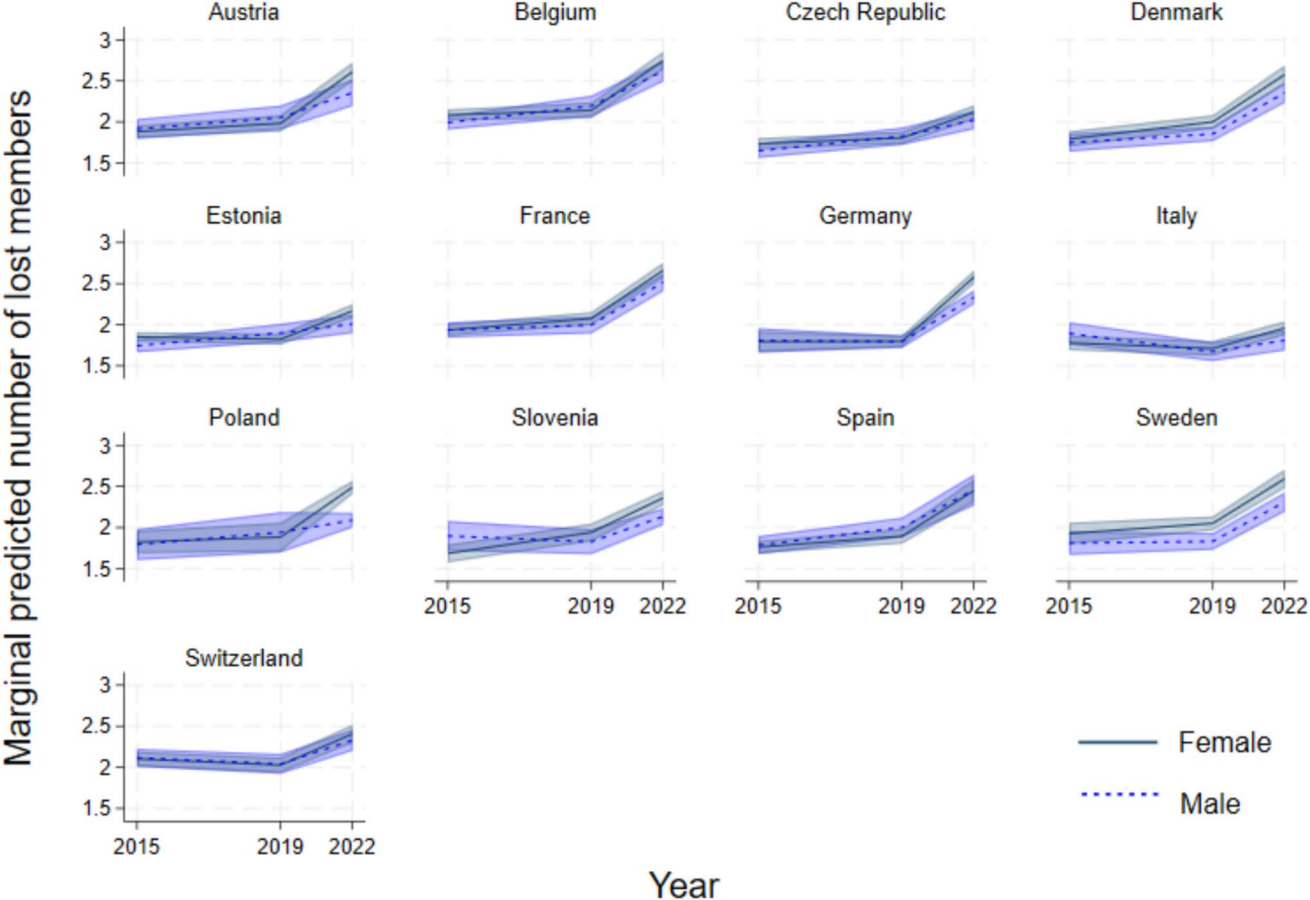
Predicted margins of the number of lost members in social networks in comparison to the previous wave based on country- and gender-specific mixed models accounting for panel attrition. *Notes*: since the models are looking at changes between the waves, each year refers to the end of the period, i.e. 2019 indicates the four-year period between 2015 and 2019^.^

While the increasing trend in social network size appears to only marginally be affected by the pandemic, and in various directions depending on the country, it is possible that these analyses are hiding important changes in social ties. Individuals may be losing or gaining new confidants, while the number of confidants they have may remain the same. We thus consider the number of social network members lost between each survey wave (Fig. 3), counting the ties mentioned in the previous wave but not in the present wave. Over the pandemic years, a short three-year period between 2019 and 2022, across the European countries in our analysis, there was a dramatic increase in older adults losing confidants, on average almost 2.5 lost members, as compared to the loss of members between 2015 and 2019, and between 2011 and 2015. Similarly, in our robustness test modelling only respondents who participated in all survey waves, there is an increase in the number of confidants lost (Appendix Fig. 9, panel d). This is quite a striking change since the time between the surveys over the last two waves is shorter than the four-year windows between the previous surveys, especially as close to 50% of interviews of wave 8 were in 2020, and roughly 20% of interviews in wave 9 were in 2021 (Appendix Fig. 8). In only a few countries, like Italy and Estonia, is the increase in the loss of confidants during the pandemic statistically insignificant.

In Denmark, Germany, Poland, Slovenia and Sweden, women lost more confidants than men during the pandemic period, losing 2.5 members on average (all else equal). Moreover, we find that this large increase in losing social ties over the pandemic is apparent in all age groups, although with some differences. In a set of age- and gender-specific mixed models, with all countries combined to ensure sufficient observations, we find that 50–64-year-olds lost social ties over the pandemic to a larger extent than 65–79-year-olds, and even more than those age 80+ (Appendix Fig. 10). In this younger age group, we also find that women were more likely to lose confidants than men.

**Fig. 4:**
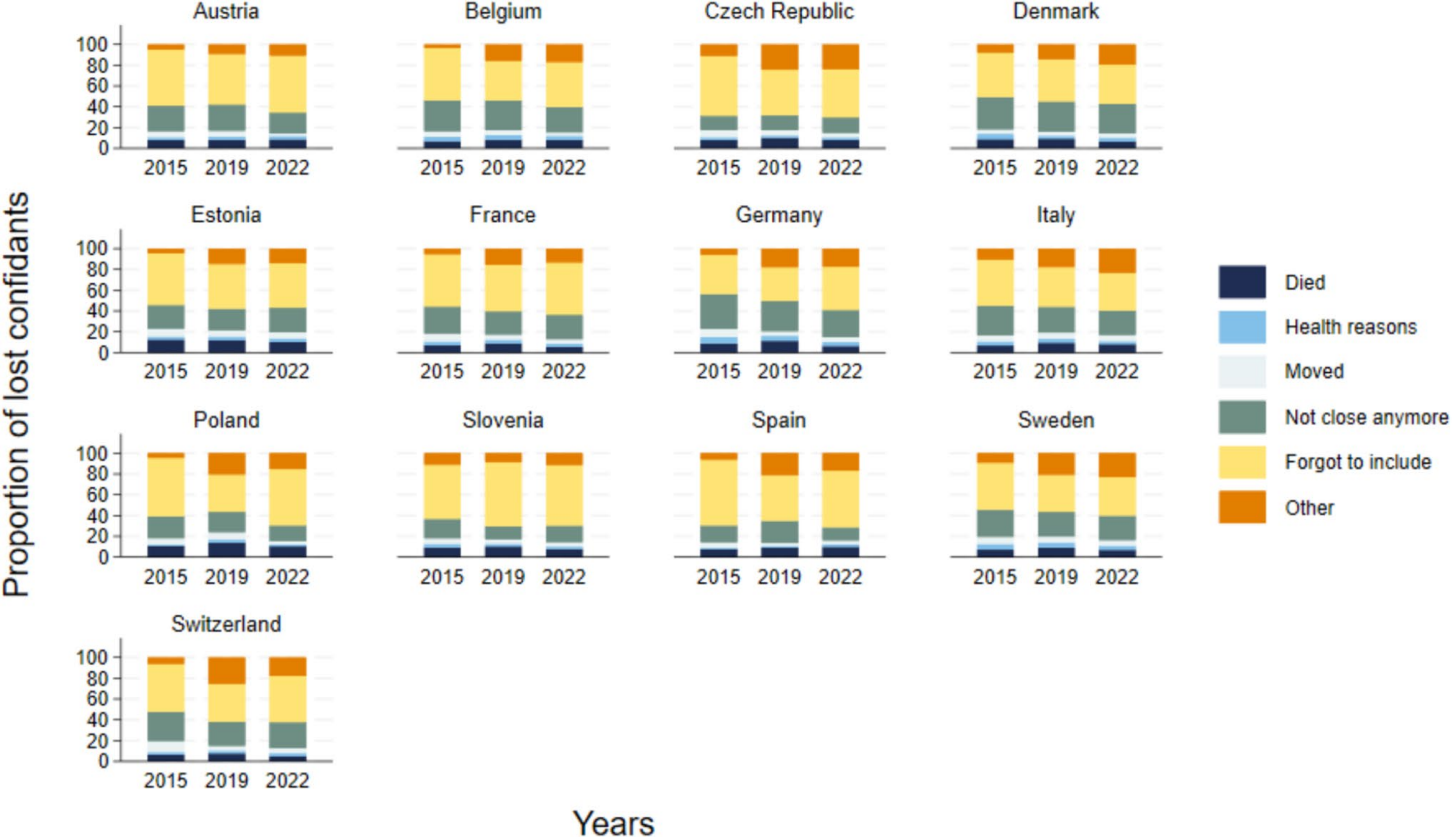
Reasons for no longer including previously named confidants in social network, as a proportion of respondents noting specific reasons

Fortunately, the data also allows us to examine why members are dropped as confidants, and not included in social networks. With the significant effect of COVID-19, increasing deaths among older adults in particular in Europe (O’Driscoll et al. 2021; Torres et al. 2023), it is possible that the loss of members of social networks is due to deaths of confidants. On average across countries, among those who provided a reason for losing a confidant, 8% attributed it to death over the pandemic period. However, death also accounted for around 8%–10% of lost members between the earlier waves (Fig. 4). Therefore, an increase in deaths does not appear to explain the increase in lost network members.

The main reason that respondents do not name confidants whom they previously noted is that they simply forgot to include them, as seen in Fig. 4. This suggests recall could cause errors in the data, but by including this option in the questionnaire, it is possible to correct for potential mistakes of forgotten ties. That said, forgetting to include a confidant may be a true reflection of this tie becoming less important to the respondent. Another key reason that confidants are lost is simply that they are no longer in touch, or as close as they were before. Roughly 30% of older adults in Austria, Belgium, Denmark, France, Germany, Italy, and Switzerland note “not being close anymore” as a reason for losing confidants – and this proportion has slightly declined over time. Unidentified “other” reasons seem to have increased over the pandemic period, as evident in Italy and Sweden. Although we are unable to unpack this, we could speculate that physical distancing policies may have prevented people from seeing each other as frequently as they would have in the past (even if they still feel close), or that fear of contracting the coronavirus amongst older adults may have limited contact.

### The effect of the pandemic on new social ties

**Fig. 5:**
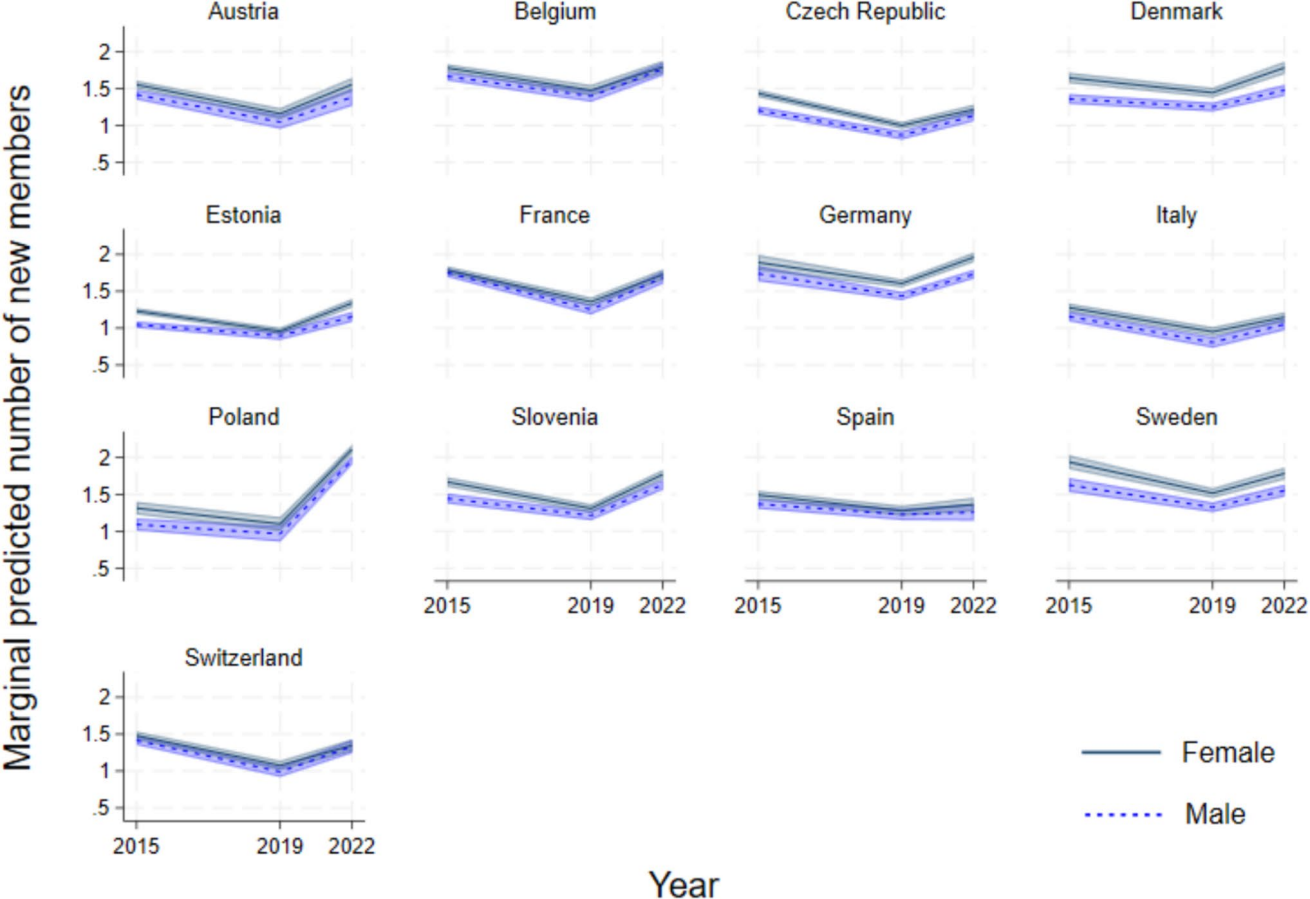
Predicted margins of the number of new members in social networks in comparison to the previous wave based on country- and gender-specific mixed models accounting for panel attrition. *Note*: since the models are looking at changes between the waves, each year refers to the end of the period, i.e. 2019 indicates the four-year period between 2015 and 2019.

Just as individuals may lose social ties, it is possible they gain new ones. In Fig. 5, we examine the addition of new members and find a U-shaped trend across all countries. Between 2011 and 2015, and over the pandemic period (2019–2022), individuals were more likely to have new confidants (roughly 1.5 new confidants), as compared to the 2015–2019 period (when the number of new confidants was closer to one). In most countries, there was an increase in the number of new members over the pandemic period, particularly apparent in Denmark and Slovenia, where the model predicted the number of new members was even higher than between the first two waves. That said, in our model of respondents interviewed in all four waves, we find that the increase in new confidants during the pandemic period is not large (Appendix Fig. 9, panel c). However, this could be related to the ageing of these respondents (they are all 10–11 years older in their last interview as compared to their first interview). When we consider the addition of new members to the social network, we find that 50–64-year-old adults are more likely to have gained new confidants over the pandemic period, close to two new confidants, which is even more than in earlier periods. Moreover, the gap between men and women is wider—with women having more new members during the entire period covered. In older age groups, we also see an uptick in new members during the pandemic but with no differences between men and women and fewer new members (Appendix Fig. 11).

**Fig. 6:**
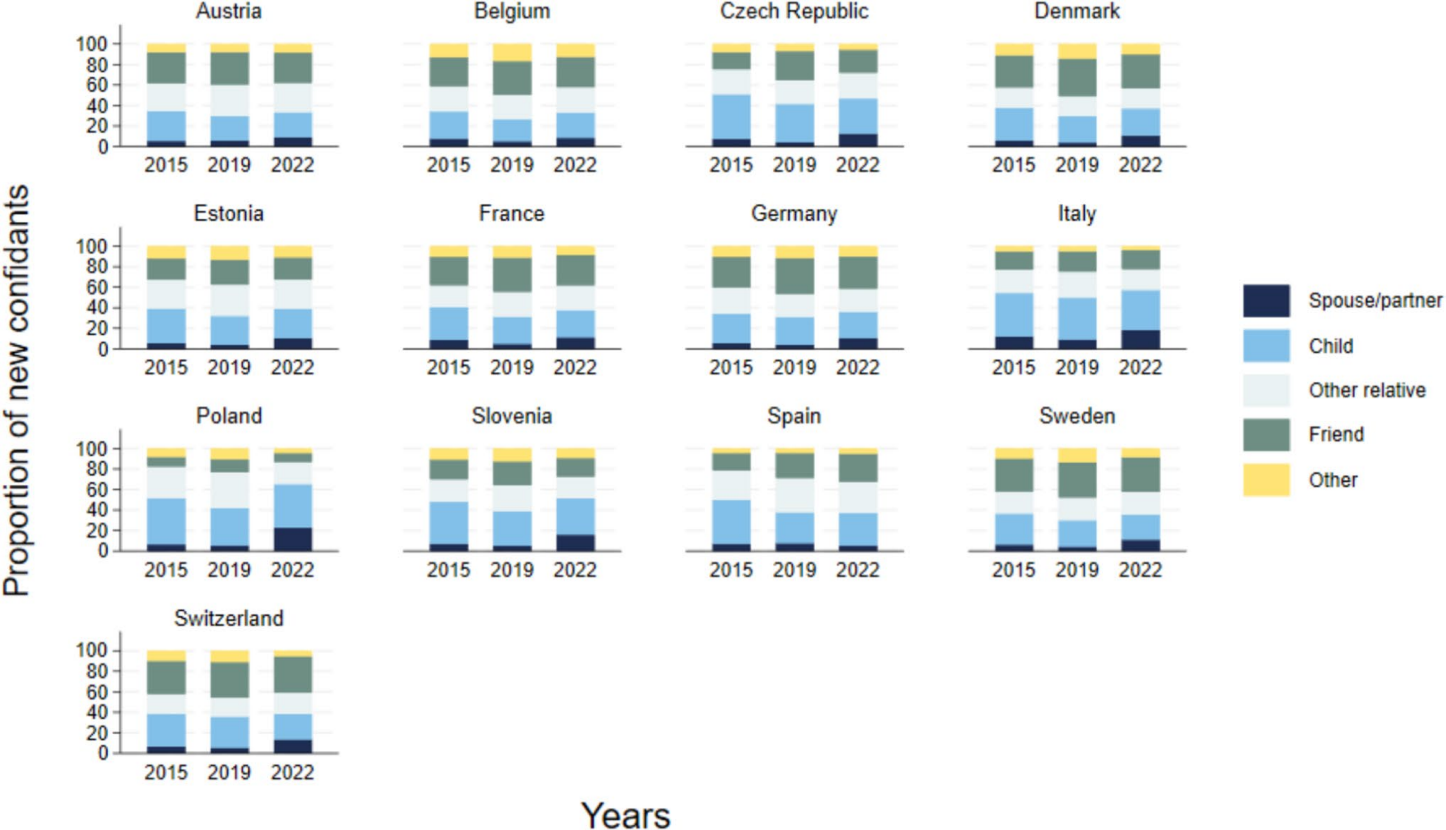
Relationship with newly named confidants in social network, as a proportion of respondents noting new members

Overall, Fig. 5 suggests that pre-pandemic there was a decline in older adults with new confidants, but over the pandemic period, there was a rebound, with people again finding new confidants. It is worth noting that an additional member to the close social network does not necessarily mean the network is expanding, since new confidants may be replacing those lost. It also does not mean that these confidants are completely new social ties, but could have previously existed, with the quality of the relationship changing to the extent that the tie is now named a confidant. In Fig. 6, we examine who these new confidants are. Indeed, we find that children, friends, and other relatives make up a large proportion of new confidants across all waves. This suggests that the quality of the social tie changed, or that having lost a different confidant, the respondents “promoted” someone new to “confidant status”. During the pandemic period, the proportion of new members who were spouses or partners increased, especially noticeable in Poland, Italy and Slovenia. The quality of the relationship with spouses may have changed, or individuals who were not in a relationship may have started one during the pandemic period, seeking companionship during the crisis period.

### Spouses as integral members of social networks in older ages

Considering the relatively low mean number of confidants older adults have, spouses (or partners) are found to be central confidants. On average over time and across countries, 61% of individuals with at least one confidant indicated that their spouse is part of their social network, and 15% have only spouses as confidants (and no one else). Over the pandemic period, there was a slight decline in having a spouse as a confidant in a handful of European countries, notably Austria, Czech Republic, France, Spain, Sweden and Switzerland (Appendix Fig. 12). This is preceded in the pre-pandemic period by a slight increase in the proportion of social ties being with a spouse.

**Fig. 7:**
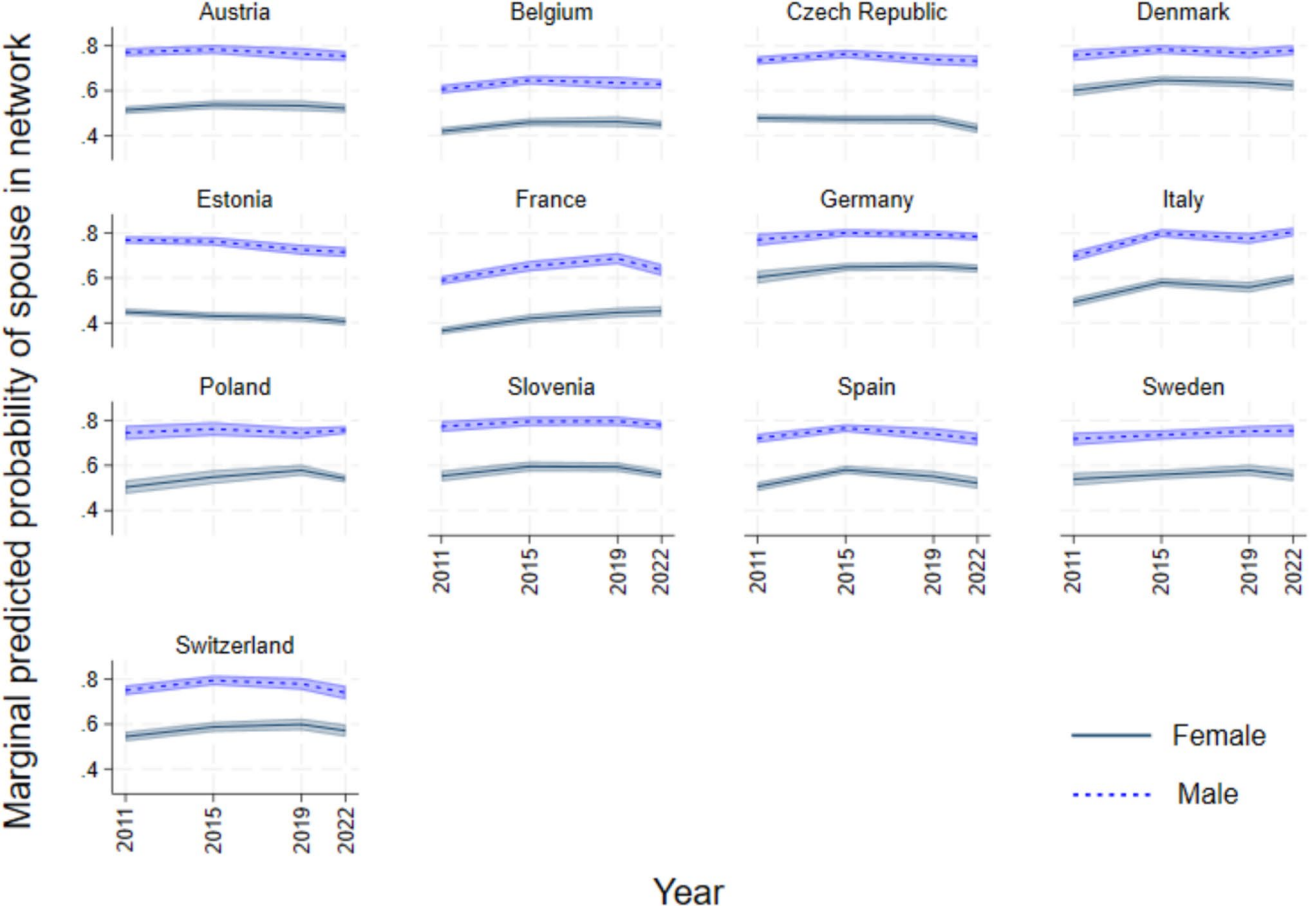
Predicted margins of the presence of a spouse in the social network based on country- and gender-specific mixed models accounting for panel attrition

When considering the presence of a spouse in older adults’ social network, within a multivariable framework accounting for age, retirement status, chronic illnesses and place of residence (as well as panel attrition using inverse probability weighting), we find that older men are much more likely to have a spouse as a confidant than women (Fig. 7). This is likely explained by the gender gap in mortality in older ages. Moreover, in most countries we examine, there is a mostly flat, somewhat inverted-U ﻿shaped association between the presence of a spouse and year.^12^ In other words, there is a slight increasing then declining trend in having a spouse as a confidant, most clearly seen in Spain and Switzerland.^13^ In Italy and France (for women), there is an increase in older adults who report spouses in their social networks over time. The pandemic seems to have some association with the inclusion of a spouse in social networks, although not statistically significant, in Switzerland and France (among men) and the Czech Republic and Poland (among women), whereby the proportion of spouses reported as confidants declined.

## Discussion

Social ties are important throughout the life course, and stronger, emotionally connected ties are prioritized by older adults (Carstensen et al. 1999; Schwartz and Litwin 2018). Interestingly, we find that new confidants of older adults are mostly children and other relatives, that is, people who are already part of their network but were not previously considered as those with whom they discuss important things. We find that over a decade, social networks of confidants increased in size amongst older adults. In parallel, we find a considerable increase in gains and losses of social ties across all waves. On average, across the countries in Europe we examine, individuals gained at least one new confidant (1.37) and lost two confidants (2.06) every three to four years (between survey waves). While more losses than gains may seem contradictory considering the overall growth in network size (see Table 2), these averages conceal individual-level dynamics. For instance, it is possible that those losing more confidants in the first place have larger networks, and those gaining new confidants have smaller networks to begin with, and the balance between them has changed over time (increasing network size).^14^ That said, it is also possible that the higher number of lost confidants reflects recall bias and/or respondent fatigue.

During the pandemic, we find that network size remained stable in most countries, or even increased in some cases, partially supporting hypothesis H1a. The relative stability contradicts studies showing a decrease in social network size, especially for older ages (Dávid et al. 2023; Haggerty et al. 2023; Wong et al. 2023). One reason for the discrepancy with our results is that prior studies primarily refer to any type of contacts, while our study considers a confidant network, which is intrinsically more stable. Moreover, previous studies on the pandemic and confidant social networks have focused on the general population (Bierman et al. 2021), whereas our study specifically examines older adults.

Moreover, in the pandemic period, the changes in social networks intensified: with an average of 1.68 gained and 2.37 confidants lost. These escalated changes are even more noteworthy considering the shorter period between surveys, and that the mean size of social networks is between two and three (Table 2). Our country- and gender-specific models of network size, which control for age, residence, retirement status and chronic illnesses, and deal with panel attrition, confirm these findings: the COVID-19 pandemic brought with it increased changes to social networks amongst older-aged adults in Europe. There was considerable churning of social networks at the time of the pandemic that derived not only from losing members but from gaining new ones too, supporting our hypothesis H3. Over the pandemic period, between 2019 and 2022, only 5.7% of older adults in our analysis did not lose or gain any confidants (ranging from 1.8% in Poland to 10.7% in Switzerland).

We find that the reasons for losing confidants over the pandemic period are not very different from the reasons mentioned in earlier years. For respondents, it is not because of increases in deaths or changes in health that more confidants are lost, despite the potential for substantial changes in health during the pandemic. Nevertheless, there may be other pandemic-related reasons that are not directly mentioned in the surveys. Notably, more individuals declared losing confidants for “other” reasons, which may include physical distancing (sometimes even self-imposed when governmental restrictions are lifted) or even arguments about the pandemic and its management. Stringency policies prevented people from maintaining close ties. Fewer possibilities of meeting in-person most likely redefined some relations, with confidants becoming less close for “other” reasons. The frequency of social contacts was reduced over the pandemic, and these effects also lingered (Wong et al. 2023), especially amongst poorer individuals (Haggerty et al. 2023). Thus, close ties may have taken time to be renewed. Regular interaction, sustained over long periods of time, is needed to maintain close relationships, especially friendships and parent-child relations (Farooqi 2014). An additional factor that may have caused the loss of confidants for other reasons may be disagreements. During times of crisis, such as the COVID-19 pandemic, conspiracy theories escalate, institutional trust weakens, and individuals may feel marginalized, undermining social ties (Thoresen et al. 2021; Freeman et al. 2022; Pummerer et al. 2022). Finally, social ties over the pandemic may have been lost since employment and economic status changed for many: working-aged older adults may have lost their jobs or had to work at a distance, losing contact with colleagues, older-aged adults were nudged towards retirement, savings for retirement were spent to finance current consumption (Chłoń-Domińczak and Holzer-Żelażewska 2022; Panarello and Tassinari 2022), and such changes may have strained some relations.

Regardless of the reason a confidant is lost, whether by choice or circumstance, the consequences of lost relations can be particularly far-reaching, whether negative or empowering (Settersten et al. 2024). Lost social ties may result in lost emotional support, lost resources, broken bridges to others and lost affiliation with certain groups. In the case of lost spouses, as we noted in some countries, women appear to be more vulnerable. Women generally have higher life expectancies, increasing their chances of losing a spouse. Since women had a lower COVID-19 burden than men (Patwardhan et al. 2024), they were particularly likely to lose spouses during the pandemic. Widowers whose spouse died of COVID-19 have faced higher mental health risks (Wang et al. 2022b). All the same, not all lost social ties are accompanied by negative outcomes. Sometimes they may signify positive or liberating changes (like in severing problematic relationships), redefinition of the self and development of independence.

Similarly, new social ties can have significant positive effects, increasing emotional and physical support and improving well-being (Eriksson et al. 1999; Himes and Reidy 2000). We find an increase in new social ties during the pandemic period, probably facilitated by digital technology enabling alternatives to face-to-face contact. Close ties could be sustained, typically with existing digital media (in fact, mostly home phones), but also with an increase in video calls (Sixsmith et al. 2022). Older adults who already had social networks maintained over many years were more likely to incorporate digital tools with the aim to continue maintaining these ties (Schafer 2024). Conversely, we note an increase in new confidants who are spouses over the pandemic period, suggesting that the crisis and the sheer scale of uncertainty that accompanied it may have pushed partners closer together, especially when other relations were forced to change due to policy restrictions. These descriptive findings seem contradicted by the multivariate analysis, which shows that generally the presence of a spouse in the confidant network was neither more nor less likely during the pandemic in most countries, thereby leading to the rejection of our hypotheses H4a and H4b. That said, there is some support for H4b (a decline of spouses as confidants) in the Czech Republic, Poland, Sweden and Switzerland. An intriguing explanation for these mixed results (stability, increase and decline) might relate to the possibility that gains of spouses as confidants may be offset by losses, resulting in no net change in probability to have the spouse in the confidant network for most countries, while in other countries (Czech Republic, Poland, Sweden and Switzerland) losses might outweigh gains of spouses as new members in the confidant network. Not only death, but some severe illness of the spouse, like COVID-19 or some post-COVID-19 conditions, including cognitive deficits (Hampshire et al. 2024; Qi et al. 2024), may have made it more difficult for the spouse to remain a confidant. It is likely that such circumstances may have been captured under the response option ‘other reasons’ for losing a confidant, rather than under the response option ‘health reasons’, since respondents might consider it unwarranted to exclude the spouse from the confidant network as a consequence of his or her health conditions.

In some ways, our findings point to strong gender gaps in social networks in older ages, and in other ways very small gaps. Notably, the size of the network is higher amongst women across countries by roughly a mean of “half a confidant” when controlling for other factors, throughout the period we examine, in line with previous studies (McDonald and Mair 2010; Fischer and Beresford 2015). Only in Italy is there no gap, and in Spain, France and Poland, the gap is negligible. Likewise, across countries, men were more likely to include a spouse in their social network. However, when it comes to new and lost members, there are minor and not statistically significant differences between men and women, with few exceptions. In Sweden, women lost confidants more than men at all times, but at the same time, they also gained new confidants more than men: overall leading to increased turnover amongst women. This could reflect women’s larger and more diverse social networks, and/or the country’s strong welfare provisions and gender-equal norms which facilitate active maintenance of social ties. Moreover, the increase in lost network members during the pandemic was greater among women in Poland, Germany, Denmark and Slovenia than amongst men.

In considering the pandemic effect differentially by country, we were also able to point to key differences between states, as well as commonalities. General increasing trends in network size were noted in all countries. However, over the pandemic, we find in Poland a decline in size, supporting hypothesis H1b, and possibly related to higher excess mortality later in the pandemic (2022) (see Table 1). In contrast, in Denmark and Estonia, steeper increases in size than during earlier periods support hypothesis H1a. In losing confidants, Estonia and the Czech Republic stand out with no clear pandemic effect, unlike in the rest of the countries. This could be due to their mixed welfare state systems of Bismarckian style with Scandinavian style, and the reforms made, including those related to health expenses and social taxes (Bob 2000). In gaining new confidants, we similarly find that over the pandemic there was only a minor increase in Spain and Italy. The strong stringency policies, especially in Italy, may have hindered older adults from making new friends or strengthening ties with family members. We find important differences across countries in naming spouses or partners as one of their confidants: in Germany and Denmark, 70% of respondents included a spouse in their network, while in Estonia and Belgium, just over half of the respondents named spouses, possibly due to the particularly high divorce rates in these countries (Sobotka and Toulemon 2008). Nonetheless, we conclude that the country variations we find are quite minimal, as the social networks' size, lost members, new members, and inclusion of spouse broadly followed the same patterns across countries over the pandemic. That said, we acknowledge that the countries with four waves of SHARE data may be a select group of countries. Indeed, Eastern European post-Soviet states (e.g. Lithuania, Georgia) are under-represented, as are poorer Mediterranean states (e.g. Greece, Albania). The countries we focus on lean towards stronger welfare state characteristics, and cannot be generalized to Europe at large.

Our findings provide partial and nuanced support for hypothesis H5, which predicted variation in network stability according to COVID-19 policy restrictions, welfare state support, and trust in institutions. The cross-national variation we find in network churn partially aligns with this; for example, older adults in Poland—a country characterized by high stringency, limited welfare provision, and moderate trust—lost an average of ~2.5 confidants, consistent with a high-instability context, whereas Denmark and Sweden, with relatively lenient measures, strong welfare provision, and high institutional trust, experienced moderate losses alongside growth in network size. However, some results complicate a straightforward corroboration of H5. Italy and Spain, also classified as high-stringency, low-support, and low-trust, showed statistically insignificant increases in lost confidants, while Switzerland—a theorized low-instability context—exhibited both increased losses and a decline in spousal ties. Moreover, the minimal variation across countries and under-representation of weaker welfare states indicate that the pandemic shock produced widespread network churn that transcended institutional boundaries.

Overall, our results point to an association between the COVID-19 pandemic and the (in)stability of social networks amongst older adults in Europe, by comparing pre-pandemic changes with changes between 2019 and 2022 in social ties. Among older adults, we find that social network size steadily increased over a decade and was not affected by the pandemic. However, there was considerable churning in the networks, which intensified over the pandemic: the pre-pandemic increasing trend of losing confidants was accentuated, and the declining inclination to have new confidants pre-pandemic was reversed. Taken together, we find greater instability of social networks among over-50-year-old adults during the pandemic. Moreover, our country- and gender-specific analysis suggests that social ties over the pandemic were largely comparable despite some minor variation across gender and across country, likely explained by socio-cultural factors, variation in stringency policies, and in excess mortality.

Nonetheless, these results should be interpreted with caution due to some limitations. The first limitation is related to our measure of social networks. We focus only on confidants, rather than the full social network. Other ties, with non-confidants, are also important (Small et al. 2024). Moreover, our measure does not distinguish between in-person and remote relations, two modes of interaction that could potentially have a different impact on well-being. However, existing findings remain inconclusive about the link between the mode of social interaction and well-being (Litwin and Levinsky 2022; Sommerlad et al. 2022). In addition, the motivation for using remote communication appears to be significant: it tends to support well-being when the purpose is to maintain social relationships (as is often the case among older adults), and it may contribute to psychological distress when used primarily for entertainment or as a means of coping with loneliness, as observed more frequently in younger adults (Bonsaksen et al. 2021; Ragnhildsløkken et al. 2024). These findings appear to mitigate the limitations of using a single, undifferentiated network measure that combines both in-person and remote contacts in research on older adults. In addition, it appears that at the time of the pandemic, only one fifth of older adults increased remote contact with family or friends (Hawkley et al. 2021). This again mitigates the limitations of combining both in-person and remote contacts.

An additional limitation is that we cannot ascertain the mechanisms associating the pandemic with the changes in social networks. In particular, what during the pandemic period caused social networks amongst older adults to change more than before? Related to this, we do not consider the exact date of the interview in relation to the pandemic—the stringency policies or the mortality. Part of the reason we do not do this is that confidant reports are not specific to particular months but cover a period of twelve months. Moreover, since the pandemic was rapidly evolving, differentially in each country, it would make little sense to relate social networks, which are slower to change, to particular points in time. Instead, by using the survey wave in our model, we cover a good chunk of the pandemic period (October 2020 to September 2022—see recall period explanation around Fig. 1 and Appendix Fig. 8). Nonetheless, we are not able to identify whether it was excess death, stringency policies or other factors that determined the changes in social networks over this pandemic period.

Final limitations call for caution in the interpretation of the results, since panel surveys can suffer from non-response and attrition, biasing the results. Respondents who are no longer included in the survey may represent a certain type of individual (of course including those who have died), or have particular social networks. This could especially be a problem if those lost to follow-up between the 2019 and 2022 survey waves are different from those lost between previous survey waves. We minimize the attrition bias by using inverse probability weights in our models. However, on top of this attrition, non-response to questions on confidants, the name-generating survey module, can be quite high, particularly amongst older individuals and women (González et al. 2024). The relatively low network size we find may be a true reflection of social networks in older ages, not limited to questionnaire restraints, or it could be a means for the respondents to reduce the burden of questions they need to answer (Stadel and Stulp 2022). Moreover, it is possible that the pandemic may have affected how people report on their social ties in the survey. Despite these limitations, the quality of the surveys and rigorous checks of names of confidants between each survey wave likely reduce the likelihood of bias.

Despite these limitations, this study represents the first longitudinal and comparative analysis of European countries focussing on the confidant networks of individuals aged 50 and over, considering the interplay between network size, gains, and losses. Some pre-pandemic long-term trends have been identified: an overall growth in network size, a declining propensity to add new members, and an increasing likelihood of losing confidants. The COVID-19 global crisis accentuated the pre-pandemic trend of confidant loss but also increased the likelihood of adding new members—thus reversing the earlier trend. Overall, this led to considerable churning within confidant networks, the consequences of which may be explored in future research. Future studies may also consider the wider social network, rather than only those with whom important matters are discussed, and whether they have recovered after the pandemic, as well as whether our findings are valid for populations outside of Europe or for other age groups. Studies unpacking how and to what extent characteristics of social ties, including interaction, emotional closeness and physical support among older adults changed over the pandemic are also warranted.

## Figures and Tables

**Fig. 8:**
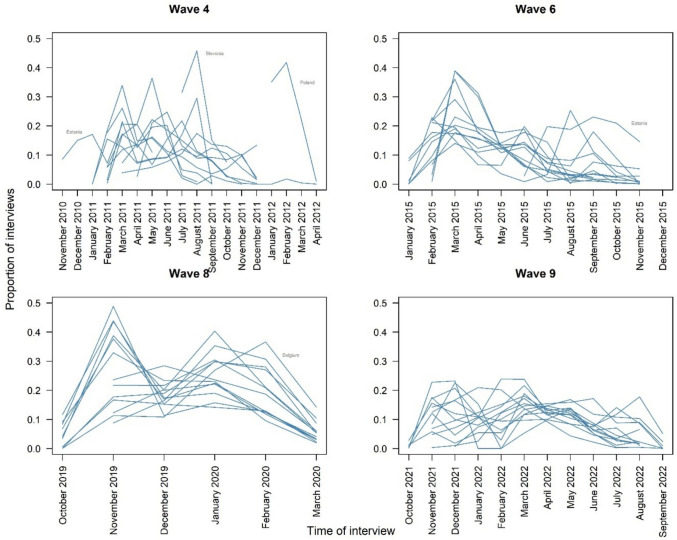
Distribution of interviews over time in each country by wave

**Fig. 9:**
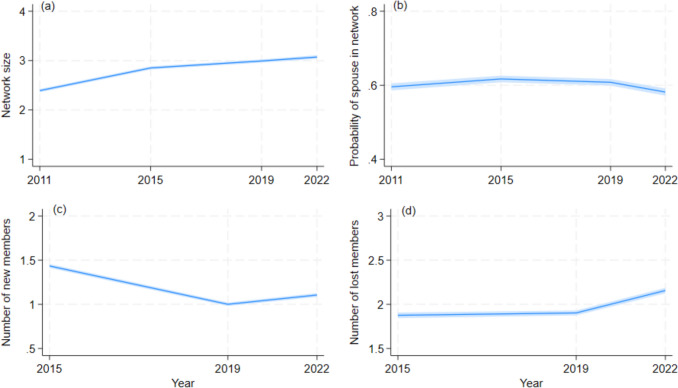
Marginal predicted effect of time on characteristics of older adults’ social network, pre-pandemic (2011, 2015 and 2019) and during the COVID-19 pandemic (2022), amongst respondents who participated in all four survey waves, based on pooled countries and for both genders. *Note*: models are for all countries combined, only with respondents who participated in all waves of the survey (25% of sample). Since the models in c and d are looking at changes between the waves, each year refers to the end of the period, i.e. 2019 indicates the four-year period between 2015 and 2019. The y-axis range is comparable to those of figures above for each outcome, e.g. panel b corresponds to Figure 7.

**Fig. 10:**
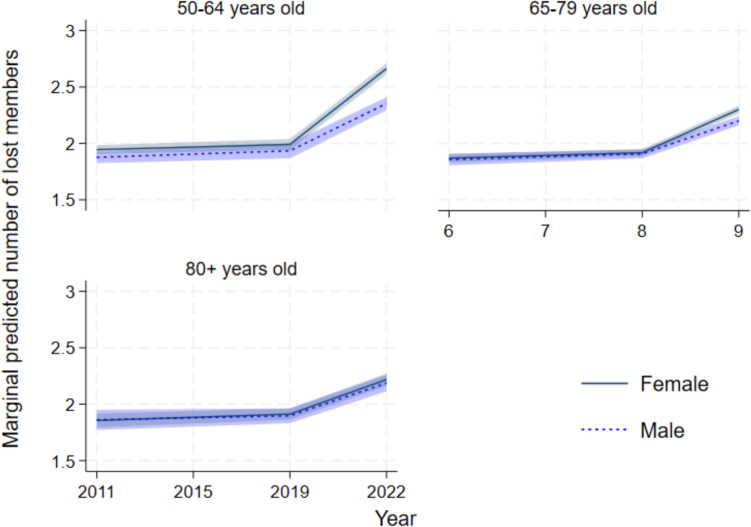
Predicting the number of lost members in social network in comparison to previous wave: Coefficients and confidence intervals of survey wave from age group and gender-specific mixed models accounting for panel attrition. *Note*: models are for all countries combined. Since the models are looking at changes between the waves, each year refers to the end of the period, i.e. 2019 indicates the four-year period between 2015 and 2019.

**Fig. 11:**
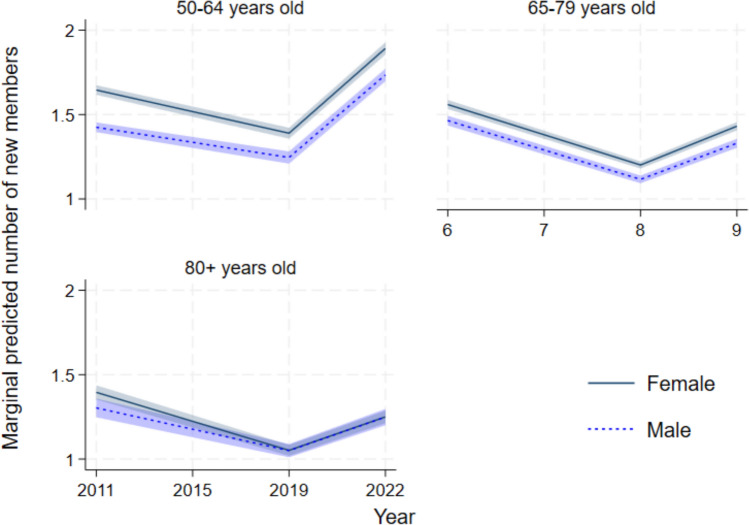
Predicting the number of new members in social networks in comparison to the previous wave: Coefficients and confidence intervals of survey waves from age group- and gender-specific mixed models accounting for panel attrition. *Note*: models are for all countries combined. Since the models are looking at changes between the waves, each year refers to the end of the period, i.e. 2019 indicates the four-year period between 2015 and 2019.

**Fig. 12:**
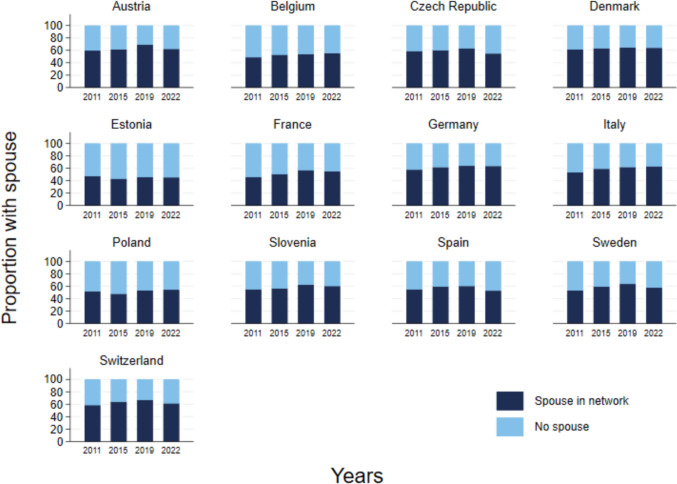
Presence of a spouse in older adults’ social network, pre-pandemic (2011, 2015 and 2019) and during the COVID-19 pandemic (2022).

## Data Availability

All data used in this manuscript came from the Survey of Health, Aging and Retirement in Europe (SHARE), available for download on request at: https://share-eric.eu/data/ We used the following data releases: Wave 4 (10.6103/SHARE.w4.900), Wave 6 (10.6103/SHARE.w6.900), Wave 8 (10.6103/SHARE.w8.900), and Wave 9 (10.6103/SHARE.w9.900).

## Appendix

See Figs.

## Footnotes

1 https://share-eric.eu/data/

2 The monthly distribution of interviews in each wave over the period covered is in Appendix Figure 8.

3 Six other countries participated in three out of the four waves: Croatia (not in wave 4), Greece (not in wave 4), Israel (not in wave 4), Luxemburg (not in wave 4), Netherlands (not in wave 6), and Portugal (not in wave 8). Since most of these countries do not include wave 4, we would have indicators of lost and new members only for the last two waves. Eight countries only participated in the more recent waves (8 and 9). We therefore found it was insufficient to provide a longitudinal perspective using less than four waves.

4 The full question is worded as follows: “*Now I am going to ask some questions about your relationships with other people. Most people discuss with others the good or bad things that happen to them, problems they are having, or important concerns they may have. Looking back over the last 12 months, who are the people with whom you most often discussed important things? These people may include your family members, friends, neighbours, or other acquaintances. Please refer to these people by their first names*.”

5 On average across countries, close to 12% of confidants are not friends or family, but include relations such as neighbours, colleagues, therapists, care providers, or religious figures.

6 Since stringency policies were being introduced already in March 2020, it is possible that the respondents interviewed during this month were experiencing changes in their social networks due to the rapidly evolving situation. In comparing the key indicators we use of social network, we find that those interviewed in March 2020 on average had a slightly smaller network size (2.6 as opposed to 2.8 among those interviewed beforehand), and were slightly more likely to have a spouse in their network (65% as opposed to 61%). Respondents interviewed in March lost and gained equivalent numbers of network members to those interviewed in the months before (1.9 lost and 1.0 new). However, it is also worth noting that only 2,807 respondents were interviewed then – 7.3% of wave 8 respondents.

7 Hot-deck imputation replaces the missing values in one or more variables for a non-respondent with the observed values from these variables obtained from a respondent who has similar characteristics.

8 Since our outcome variables are counts or dichotomous, we also tested Poisson and Logit models with individual fixed effects, but which do not account for attrition. Results were robust, though more conservative.

9 To calculate the weights, we estimated a series of logistic regression models predicting inclusion of respondent in each wave, as a function of the independent variables, conditional on participation in the previous wave (Wooldridge 2002; Tchetgen et al. 2012). For each observation we computed the inverse of the predicted probabilities from these models and used them to weight each observation in the analysis.

10 We ran a series of Haussman tests to check whether it was necessary to use fixed effects models. To account for heteroskedasticity, we use the “robust” option for more precise standard errors.

11 We considered using life expectancy to account for the effect of the COVID-19 pandemic at the country level. We used life expectancy rather than excess mortality since excess mortality is essentially zero in the pre-pandemic years (which are used as the benchmark). Period life expectancy captures well the increase in deaths at the time of the pandemic, including the differences across countries (Schöley et al. 2022; Goldstein and Lee 2024). Using data on annual life expectancy from the World Bank (World Bank 2024), we compared country-specific models with and without life expectancy as a covariate. Life expectancy seemed to reduce the effect of wave. However, we were not confident in including life expectancy in the models since in some countries the correlation between wave and life expectancy was high. Moreover, life expectancy at the time of the pandemic (2020 and 2021) is less evident since we have survey waves only before and after, and some countries recovered quickly so we miss the pandemic effect.

12 This is similarly seen in a pooled model of only respondents who participated in all waves of the survey (Appendix Figure 9, panel b).

13 Due to the scale of the y-axis in Figure 7, the more refined changes including the inverted-U shape are less evident.

14 Model coefficients of network size in predicting new members indicate that a larger network significantly increases the likelihood of a new member (by about 70% across countries for men and women alike). However, in predicting lost members, network size is not found to be significant in most countries, and the effect is smaller: in Germany, Denmark, Poland and Slovenia, a larger network is associated with increased odds of losing a member (for men and women), but amongst women in Sweden and Switzerland there is a small negative relationship between network size and losing a member.
